# Rapid Detection and Identification of *Yersinia pestis* from Food Using Immunomagnetic Separation and Pyrosequencing

**DOI:** 10.1155/2012/781652

**Published:** 2012-10-03

**Authors:** Kingsley K. Amoako, Michael J. Shields, Noriko Goji, Chantal Paquet, Matthew C. Thomas, Timothy W. Janzen, Cesar I. Bin Kingombe, Arnold J. Kell, Kristen R. Hahn

**Affiliations:** ^1^Lethbridge Laboratory, National Centres for Animal Disease, Canadian Food Inspection Agency, P.O. Box 640, Township Road 9-1, Lethbridge, AB, Canada T1J 3Z4; ^2^Emerging Technologies Division, National Research Council, 100 Sussex Drive, Ottawa, ON, Canada K1A 0R6; ^3^Sir F. G. Banting Research Centre, Health Canada, 251 Sir Frederick Banting Dr., Tunney's Pasture, Ottawa, ON, Canada K1A 0K9

## Abstract

Interest has recently been renewed in the possible use of *Y. pestis*, the causative agent of plague, as a biological weapon by terrorists. The vulnerability of food to intentional contamination coupled with reports of humans having acquired plague through eating infected animals that were not adequately cooked or handling of meat from infected animals makes the possible use of *Y. pestis* in a foodborne bioterrorism attack a reality. Rapid, efficient food sample preparation and detection systems that will help overcome the problem associated with the complexity of the different matrices and also remove any ambiguity in results will enable rapid informed decisions to be made regarding contamination of food with biothreat agents. We have developed a rapid detection assay that combines the use of immunomagnetic separation and pyrosequencing in generating results for the unambiguous identification of *Y. pestis* from milk (0.9 CFU/mL), bagged salad (1.6 CFU/g), and processed meat (10 CFU/g). The low detection limits demonstrated in this assay provide a novel tool for the rapid detection and confirmation of *Y. pestis* in food without the need for enrichment. The combined use of the *i*CropTheBug system and pyrosequencing for efficient capture and detection of *Y. pestis* is novel and has potential applications in food biodefence.

## 1. Introduction

Plague, caused by *Yersinia pestis*, has given rise to three major pandemics and is considered one of the most devastating diseases in human history [1]. It still poses a significant threat to human health and remains a current threat in many parts of the world with about 2–3000 cases reported annually [2]. Due to the ease of transmission and the reappearance of plague in several countries, it has been recently categorized as a reemerging disease [3]. Furthermore, interest has been renewed in the possible use of *Y. pestis* as a biological weapon by terrorists, as it could cause mass casualties if dispersed as an aerosol [4]. *Y. pestis* is most commonly transmitted through flea bites in animals and the disease is manifested as bubonic, septicemic, or pneumonic plague [2, 5]. However, human plague has also been acquired through eating infected animals that were not adequately cooked or through the handling of meat from infected animals [6–13]. These reports demonstrate that human plague can be acquired through the oropharyngeal route and hence poses a significant public health risk. The vulnerability of food has been demonstrated by the intentional contamination of salad bars in the United States with *Salmonella typhimurium*, and this makes the possible use of more deadly agents such as *Y. pestis* a possibility [14]. This concern is exacerbated by the report of multidrug resistant strains [15] and their potential use for bioterrorism in the human population. To minimize this risk, the development of rapid detection systems that will enable the simultaneous detection and confirmation of the presence of *Y. pestis* is essential. Sample preparation and detection systems that will help overcome the problems associated with the complexity of different matrices and also remove any ambiguity in results will enable rapid informed decisions to be made regarding contamination of food with biothreat agents.

The recent development of next generation sequencing platforms has opened up new opportunities and helped change the direction of microbial genomics and its application for pathogen detection [16]. Sequencing-based technologies are becoming rapid, cost effective, and yield substantially more genetic information which helps to quickly make informed decisions on foodborne disease outbreaks. This was seen in the recent *E. coli* outbreak in Europe, where the strain implicated was sequenced in a record time of a few hours [17]. It also offers an added layer of confidence in the identification of pathogens and provides an unambiguous detection system for biodefence applications such as foodborne bioterrorism response. Pyrosequencing is a sequencing-by-synthesis method that quantitatively monitors the incorporation of nucleotides in real time, through the emission of light following the enzymatic conversion of pyrophosphate released during nucleotide incorporation [18]. This technique generates similar data to Sanger sequencing and is a rapid, reproducible, high-throughput, user-friendly, and cost-effective method [19].

We have recently developed an immunomagnetic separation (IMS) assay for the efficient concentration of *Bacillus anthracis* spores from different food matrices [20] and a novel sequence-based assay using pyrosequencing for the specific detection and antimicrobial resistance gene profiling of *Y. pestis *[21]. Here, we present the application of IMS and pyrosequencing based assays for the rapid, specific, and sensitive detection and identification of *Y. pestis* from food matrices such as milk, bagged salad, and processed meat. This assay for *Y. pestis* detection is a significant improvement over our previous work using the Pathatrix sample preparation system and real-time PCR [22] and demonstrates better limits of detection without an enrichment step. The combination of efficient immunomagnetic concentration of biothreat agents and pyrosequence-based detection system is novel and represents the first report for detection and identification of *Y. pestis* in food with potential biodefence application.

## 2. Materials and Methods

### 2.1. Bacterial Culture

*Yersinia pestis* KIM5- was cultured from a glycerol stock on Tryptic Soy Agar plates (Difco, Becton Dickinson, Sparks, MD, USA) supplemented with 5% sheep blood (TSBAP) and grown at 28°C for 48 h. A single colony was subcultured in Brain Heart Infusion (BHI) broth for 24 h at 37°C. Cultures were serially diluted in BHI, enumerated using TSBAP, and used for IMS in food and pyrosequencing experiments.

### 2.2. Magnetic Bead Functionalization with Anti-*Y. pestis* Antibodies

Two types of beads of different sizes and surface chemistries, consisting of the commercially available Pathatrix beads (~1 *μ*m diameter, Life Technologies, Carlsbad, CA, USA), and NRC-beads (300 nm diameter, National Research Council, Ottawa, ON, Canada), were used for functionalization. The Pathatrix and NRC magnetic beads were functionalized with anti-*Y. pestis* antibody polyclonal rabbit anti-*Y. pestis *(Tetracore, Rockville, MD, USA) or *Y. pestis *monoclonal Clone# M996145 (Fitzgerald Industries International, Acton, MA, USA) at a concentration of 1 mg/mL using the Pathatrix custom-coating kit with slight modifications [20]. The functionalized beads were adjusted to a final concentration of 20 mg/mL and stored at 4°C until use.

### 2.3. Comparison of IMS Methods, Antibodies, and Immunomagnetic Beads for the Capture of *Y. pestis* in Buffered Peptone Water (BPW)

The two methods for capturing *Y. pestis*, Pathatrix Auto system (Life Technologies, Carlsbad, CA, USA) and *i*CropTheBug systems (FiltaFlex Ltd., Almonte, ON, Canada), were used as previously described [20]. To compare each machine for capture efficiency of *Y. pestis,* 1 mg of Pathatrix immunomagnetic beads (IMBs) functionalized with rabbit anti-*Y. pestis *and 50 mL of BPW (pH 7.2) containing ~5 CFU/mL of *Y. pestis* were mixed for 1 h, after which the beads were magnetically captured from the solution. The two different antibodies (monoclonal and polyclonal) were used to functionalize the Pathatrix beads and investigated for sensitivity with the *i*CropTheBug system. 

 The Pathatrix beads and NRC beads functionalized with the Rabbit anti-*Y. pestis *antibody were compared against one another in the same fashion as the antibody comparison described above. One milligram of functionalized beads was mixed with 50 mL of BPW containing *Y. pestis *(~2.5 CFU/mL) and captured.

The captured beads for all experiments were washed 3 times in washing buffer, resuspended in PBS buffer, plated on Tryptic Soy Blood Agar Plates (TSBAPs), and incubated for 48 h at 28°C for colony enumeration. The experiments/assays were run in triplicate and plated in duplicate.

### 2.4. Data Analysis

Data for the IMS experiments were analysed by dividing the total number of *Y. pestis* cells captured by the total number of cells added to the BPW, and expressed as percent recovery. The total number of *Y. pestis* cells added was determined by plate enumeration of prepared stock prior to each run. Standard deviations were calculated from the mean results of the replicate experiments.

### 2.5. Preparation of Spiked Food Samples

Whole milk (3.25% milk fat), processed meat (black forest ham), and prewashed bagged salad (romaine lettuce) were purchased from a local grocery store and used for the food-spiking experiments as previously described [20]. Briefly, *Y. pestis* cultures were grown to a concentration of 10^7^ CFU/mL and serially diluted to 10^2^–10^4^ CFU/mL. Cells were added to 50 mL of whole milk to achieve a cell inoculation of 0.1–7 CFU/mL of *Y. pestis*. For bacterial capture in solid foods, 50 g of sliced black forest ham and 50 g of bagged salad were separately placed into a stomacher bag. The samples were inoculated with 0.3-1150 CFU/g of *Y. pestis *cells and hand massaged to evenly distribute the bacteria throughout the food. Fifty millilitres of BPW was added (1 : 1 dilution w/v) and the mixture was stomached with the Stomacher 400 Circulator (Seward Ltd., West Sussex, UK). The liquid was further passed through a sponge filter and a 50 *μ*m stainless steel mesh filter using a vacuum pump and the filtrate was collected for analysis.

### 2.6. IMS of *Y. pestis *from Spiked Food Samples

Following the preparation of spiked food samples, 50 mL of the prepared food sample was mixed with 1 mg (50 *μ*L) of Pathatrix beads functionalized with Rabbit anti-*Y. pestis* polyclonal antibody. The beads were mixed and captured according to the *i*CropTheBug method as previously described [20]. Experiments involving each food matrix and bacterial concentration were done in duplicate.

### 2.7. DNA Preparations from Spiked Food Samples

The preparation of DNA from samples captured from the different foods was done as previously described [22]. Briefly, 50 *μ*L of bead samples captured from food were lysed by vortexing vigorously and heating at 95°C in a thermal cycler. Following a brief centrifugation, 3.5 *μ*L of the supernatant was used as template for PCR amplification and then followed by pyrosequencing analysis. The PCR primers and reaction conditions are indicated in our previous work [21].

### 2.8. Pyrosequencing Analysis

Genomic DNA from *Y. pestis* samples isolated from food were analysed using our previously described pyrosequencing assay [21]. Briefly, biotinylated PCR products were bound to streptavidin-coated sepharose beads (GE Healthcare, Piscataway, NJ) and the beads were then resuspended in annealing buffer containing 0.3 *μ*M of the sequencing primer. Pyrosequencing was performed using the Pyro Gold Q24 reagents in triplicate, using dispensations based on the target sequence with the Pyromark Q24 system. Raw data files were imported into Pyromark Q24 software (version 2.0; Qiagen Inc. [http://www.qiagen.com/products/pyromarkq24.aspx]) for analysis following pyrosequencing. Sequence data that passed the quality check, as determined automatically by the software, were compared to the GenBank database (http://www.ncbi.nlm.nih.gov/genbank/) using the sequence search function in Geneious (version 5.3.5; Biomatters Inc. [http://www.geneious.com/]) to verify identity.

## 3. Results

### 3.1. Immunomagnetic Capture of *Y. pestis* Cells in BPW

**Figure 1:**
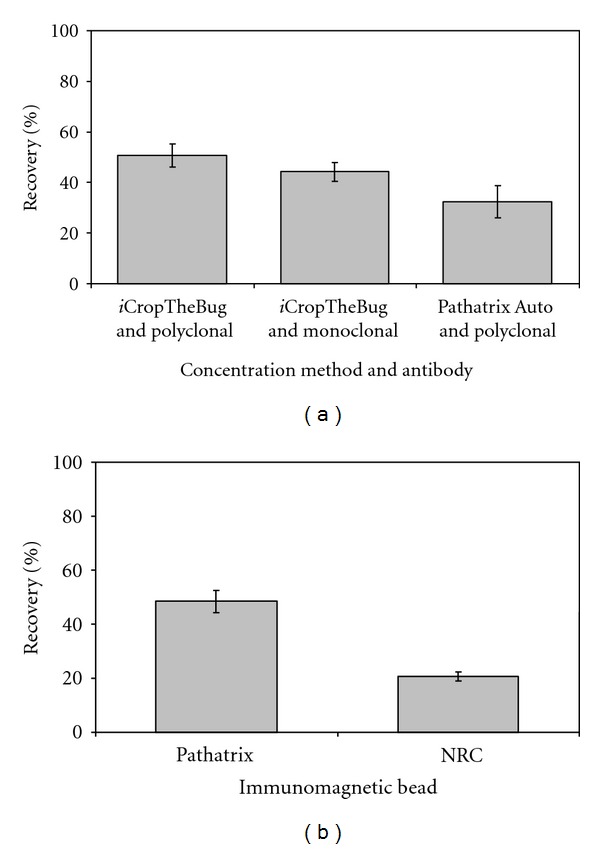
Comparison of different recovery methods, antibodies and immunomagnetic beads for the recovery of *Y. pestis*. (a) Pathatrix beads functionalized with polyclonal and monoclonal antibodies were compared for *Y. pestis* recovery using the *i*CropTheBug. The *i*CropTheBug and Pathatrix Auto systems were compared for recovery using Pathatrix beads functionalized with polyclonal antibody. (b) Pathatrix beads and NRC beads functionalized with polyclonal antibodies were compared using the *i*CropTheBug.

The polyclonal antibodies showed a better recovery of *Y. pestis*, with efficiencies of 46–56%, when compared to the monoclonal antibody with 40–48% (Figure 1(a)). The *i*CropTheBug system showed a better recovery of *Y. pestis,* when compared to the Pathatrix Auto system which had efficiencies of 26–38% (Figure 1(a)). A comparison of the two beads indicated that the Pathatrix beads were more efficient in the recovery of *Y. pestis* bacterial cells than the NRC beads (Figure 1(b)). The Pathatrix beads functionalized with polyclonal Rabbit anti-*Y. pestis* antibody, in combination with the *i*CropTheBug method, showed the most sensitive IMB/antibody combination (Figure 1).

### 3.2. Pyrosequencing of *Y. pestis *Samples Captured from Food

**Figure 2:**
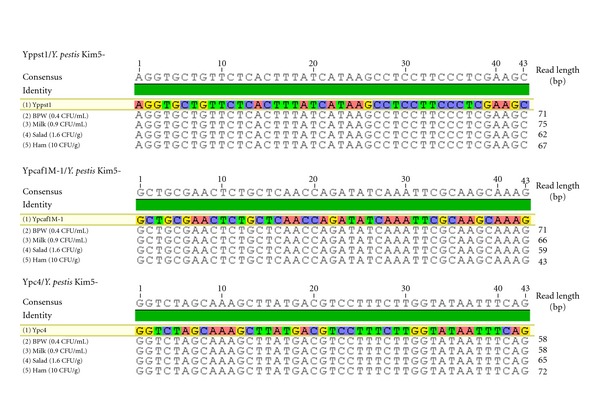
Pyrosequence alignments from *Y. pestis* isolated from food samples. Pyrosequencing reads for *Y. pestis *targets including Ypc4 (chromosome), Yppst1 (pPCP1 plasmid), and Ypcaf1M-1 (pMT1 plasmid) are shown with limit of detection from milk, ham, and salad samples.

The pyrosequencing of *Y. pestis* cells captured from the three different food matrices, conducted for targets Ypc4, Ypcaf1M1, and Yppst1, yielded reads identical to those observed in our previous report [21] (Figure 2). As previously observed, they yielded BLAST results exclusive to *Y. pestis* and thus confirmed the identification of *Y. pestis*. 

### 3.3. Limit of Detection in Food Samples

The limits of detection for the three food matrices were determined using pyrosequencing (Table 1). These results indicate detection limits of 0.9 CFU/mL, 1.6 CFU/g, and 10 CFU/g for milk, bagged salad, and processed meat, respectively. 

## 4. Discussion

The advent of novel-sequencing technologies, such as pyrosequencing, provides tools for the generation of sequence information which helps in the detection of pathogens and enables their rapid confirmation/identification. The use of these novel tools is further enhanced by the availability of whole genome sequences that provide unprecedented genetic information for the generation of specific molecular markers for diagnostic applications. These markers, if carefully selected could be used to discriminate between closely related pathogenic strains and could be used in the specific detection and identification of microbial contamination in food. The detection of *Y. pestis *in food matrices using real-time PCR has been previously reported [22], however, the limits of detection reported required further improvement. The potential for contamination with very low bacterial numbers in food matrices necessitates the development of methods for efficient capture from food matrices. The present work explored the use of a novel IMS as an efficient capture method and pyrosequencing for the detection and confirmation of *Y. pestis* directly from food without enrichment.

 The use of IMS for the efficient capture of pathogens from food has received wide attention [22–26]. There is limited information on capture and detection of* Y. pestis* in food [22] and, therefore, a need to explore this further. In a recent publication [20], we described the use of the *i*CropTheBug system as a novel immunocapture method for the concentration of anthrax spores from food. Using this same capture method, we investigated the use of different magnetic beads and antibodies for the capture of *Y. pestis* cells. Here, we show that the highest capture efficiency is associated with the use of the Pathatrix beads in combination with a polyclonal antibody (Figure 1(a)). This high capture efficiency is reflected in the limits of detection observed. The effects of bead size and different antibodies on capture efficiency have been previously discussed [20, 27–30]. Results from the study suggest that even though beads with small size present a large surface area to volume ratio, capture efficiency may be reduced due to the small magnetic core. Hence, the Pathatrix beads (which are much bigger) may possess a larger magnetic core than the NRC beads (Figure 1(b)) and thus reflect a higher capture efficiency associated with this bead type. Thus, there is a trade-off for bead size; too small is detrimental to magnetic effect, while too large appears to have some decreased capture efficiency. In our previous study on *B. anthracis*, we showed that polyclonal antibodies demonstrate a much higher efficiency in the capture of anthrax spores than monoclonal antibodies [20], however, the results obtained in the current study for *Y. pestis* suggest that the recoveries associated with the two antibodies are comparable (Figure 1(a)). The differences may be due to the antigenic targets used for the generation of the antibodies. Information to substantiate this is lacking, as they are both commercial and proprietary issues do not allow the disclosure of the antigenic targets used. Further studies using *Y. pestis* strains possessing different mutations in surface markers such as the F1 antigen are required to delineate the capture specificity of the antibodies.

Food is vulnerable to intentional contamination and the tainting of salad bars in the USA with *Salmonella typhimurium *highlights this risk [14]. There are very few sample preparation methods that do not rely on enrichment prior to detection. The IMB mixing and recovery system also play a key role in IMS. Two methods for mixing and recovery of the *Y. pestis *cells with IMB were compared. The Pathatrix Auto system is currently one of the commonly used methods for the magnetic concentration of pathogens from food matrices [22, 31], however, it had a relatively low recovery when compared to the *i*CropTheBug system (Figure 1). This is similar to what has been seen in other studies [20, 32]. 

Pyrosequencing has been used for the detection and typing of several microbes [33–35]. The pyrosequencing reads observed in the present study show consistently high sequence identities to the expected sequences, and therefore reinforce the reliability of the assays as a confirmatory tool. Typical runs were completed in about 60 minutes and hence offer a rapid sequence based detection method with unprecedented limits of detection for *Y. pestis* in a foodborne application (Table 1) [22]. In this study, all liquid matrices showed detection limits of 0.4–0.9 CFU/mL *Y. pestis *cells, while the solid matrices ranged between 1.6–10 CFU/g (Table 1). Previous work done on *Y. pestis* in milk and ground beef showed detection levels of 10^1^ CFU/mL in milk and 10^2^ CFU/g in ground beef without enrichment [22]. Previous reports indicate the limit of detection is 10^3^ CFU/mL for the IMS and detection of *Bacillus stearothermophilus* spores from food and environmental samples [36] while Shields et al. showed recovery of *B. anthracis* spores as low as 1 CFU/mL from food without enrichment. The low detection limit of the assay demonstrated in the present study represents a significant improvement over those derived from our previous work using real-time PCR [22] and provides a novel tool for the rapid detection and confirmation of *Y. pestis *in food without the need for enrichment. 

This study has further demonstrated that pyrosequencing is a proven technology for sequence-based identification and the technologyhas an unprecedented set of properties that makes it uniquely suited to, and a highly powerful tool for, biodefence applications. The technology is less expensive, time consuming, and labor intensive, as well as easier to perform than conventional Sanger sequencing [19, 37]. To our knowledge, this is the first report on the use of pyrosequencing for the direct detection from food samples. The combined use of the *i*CropTheBug system with pyrosequencing is novel for *Y. pestis* capture and detection in food and offers a new tool with an added layer of confidence for biodefence applications.

## Figures and Tables

**Table 1:** Detection limits of *Y.* *pestis* KIM5- in experimentally inoculated food matrices using IMS and Pyrosequencing.

Sample matrix	Ypc4	Yppst1	Ypcaf1M1
BPW (CFU/mL)	0.4	0.4	0.4
Milk (CFU/mL)	0.9	0.9	0.9
Salad (CFU/g)	1.6	1.6	1.6
Ham (CFU/g)	10	10	10
